# Impact of library preparation protocols and template quantity on the metagenomic reconstruction of a mock microbial community

**DOI:** 10.1186/s12864-015-2063-6

**Published:** 2015-10-24

**Authors:** Robert M. Bowers, Alicia Clum, Hope Tice, Joanne Lim, Kanwar Singh, Doina Ciobanu, Chew Yee Ngan, Jan-Fang Cheng, Susannah G. Tringe, Tanja Woyke

**Affiliations:** Microbial Genomics Program Lead, DOE Joint Genome Institute, 2800 Mitchell Dr, Walnut Creek, CA USA

**Keywords:** Low input, Low biomass, Metagenome, Library preparation protocol, Microbiome

## Abstract

**Background:**

The rapid development of sequencing technologies has provided access to environments that were either once thought inhospitable to life altogether or that contain too few cells to be analyzed using genomics approaches. While 16S rRNA gene microbial community sequencing has revolutionized our understanding of community composition and diversity over time and space, it only provides a crude estimate of microbial functional and metabolic potential. Alternatively, shotgun metagenomics allows comprehensive sampling of all genetic material in an environment, without any underlying primer biases. Until recently, one of the major bottlenecks of shotgun metagenomics has been the requirement for large initial DNA template quantities during library preparation.

**Results:**

Here, we investigate the effects of varying template concentrations across three low biomass library preparation protocols on their ability to accurately reconstruct a mock microbial community of known composition. We analyze the effects of input DNA quantity and library preparation method on library insert size, GC content, community composition, assembly quality and metagenomic binning. We found that library preparation method and the amount of starting material had significant impacts on the mock community metagenomes. In particular, GC content shifted towards more GC rich sequences at the lower input quantities regardless of library prep method, the number of low quality reads that could not be mapped to the reference genomes increased with decreasing input quantities, and the different library preparation methods had an impact on overall metagenomic community composition.

**Conclusions:**

This benchmark study provides recommendations for library creation of representative and minimally biased metagenome shotgun sequencing, enabling insights into functional attributes of low biomass ecosystem microbial communities.

**Electronic supplementary material:**

The online version of this article (doi:10.1186/s12864-015-2063-6) contains supplementary material, which is available to authorized users.

## Background

Cultivation-independent studies are revolutionizing our understanding of global biodiversity, and offering new insights into the roles that microbes play in the planet’s biogeochemical cycles. However, many microbial habitats of recent interest can be considered “low biomass” systems, as these sample types may only allow access to a few thousand cells. Such environments include the atmosphere [1], the deep subsurface [2], the built environment [3] and free viral communities [4, 5], among others. While each of these environments is physically distinct, they all require exceptionally large sampling volumes in order to obtain sufficient quantities of DNA for downstream processing. For example, many cubic meters of air are required for an atmospheric sample [6], hundreds of liters of fluids for a subseafloor sample [7], and even larger volumes for viral communities [8]. Even at these large sampling volumes, DNA extractions from low biomass samples rarely produce more than a few picograms of DNA, the equivalent of a few thousand microbial cells (1000 cells x 1 fg DNA per cell = 1 pg) [9]. While such ultra-low DNA quantities may easily enable amplicon sequencing, as witnessed by the wealth of data on the types of microbes that exist in these systems, a typical metagenomic library preparation requires as much as a microgram of input DNA [10].

The desire of the scientific community to move beyond PCR-based surveys to understand the community functional attributes in low biomass ecosystems has gone hand-in-hand with recent efforts by commercial vendors to develop low DNA template sequencing library protocols. Methods to enrich or amplify DNA from low biomass environments are presently available, however each of these has its own set of biases. For example, multiple-displacement amplification (MDA) uses phi29 DNA polymerase to produce millions of copies of template DNA and has been previously used to increase template quantities in samples prepared for metagenomic sequencing [11]. However, MDA specific biases include non-uniform coverage of MDA templates [12], shifts in GC profiles and as a result, an altered microbiome [13], yet in some environments, this bias is thought to be minimal [11]. Linear amplification for deep sequencing (LADS) [14] and linker amplification (LA) [4] have been developed to decrease some of these biases, although significant laboratory expertise and handling time are required. An interesting alternative is the MALBAC (multiple annealing and looping-based amplification cycles) method of genome amplification, which uses a semi-linear amplification to reduce amplification bias and increase genome coverage [15], however this method has yet to be tested on mixed cell populations (i.e. metagenomic samples).

Each of the methods described above requires significant laboratory manipulation, which makes production level scaling challenging. Two low template library preparation methods that may be amenable to higher throughput are Illumina’s Nextera XT kit and NuGEN’s Mondrian microfluidics workstation in conjunction with the NuGEN Ovation library preparation kit. Importantly, these kits also dramatically reduce the amount of hands-on laboratory prep time, which naturally decreases contamination risks. The Nextera XT kit uses a transposase mediated reaction that combines polishing and ligation into a single 5-min reaction. The Mondrian microfluidics system together with the Ovation library prep protocol takes advantage of microfluidics to automate many of the steps involved in a typical next-gen library preparation. Both Nextera XT and Mondrian microfluidics systems have made considerable strides towards reducing input requirements, as both kits currently recommend a minimum of 1 ng input DNA.

Nevertheless, 1 ng is still orders of magnitude higher than the amount of DNA typically extracted from a low biomass sample. Therefore, either amplification methods with minimal bias, or library preparation protocols with even lower input requirements are urgently needed. While amplification methods without bias are currently not technically realistic, reducing input requirements to levels below those recommended by the manufacturers may be possible when contaminant levels are kept low. For example, work by Chafee et al. [16] demonstrated that high quality metagenomic libraries can be constructed from as little as 50 pg of input DNA with the Nextera XT kit (Illumina), Solonenko et al. [5] generated viral metagenomes with as little as 10 pg starting material using the Linear Amplification (LA) method, and Adey et al. [17] demonstrated that the Nextera protocol can be used on just three copies of the human genome without significant coverage bias, the equivalent of 10 pg of genomic DNA.

Despite these advances, to our knowledge no study to date has systematically benchmarked and analyzed multiple library preparation protocols across a gradient of input DNA levels and tested the limits thereof. Using a defined mock metagenomic community of 26 taxa, we evaluated the performance of three distinct low DNA template sequencing library preparation protocols: the Nextera XT, the Mondrian microfluidics system and the MALBAC single- cell technology. Library preparation performance and the resulting mock metagenome sequence data were evaluated across a range of template quantities spanning 50 ng to 1 pg and compared to a 200 ng unamplified TruSeq control library.

## Results

### General library statistics

**Fig. 1:**
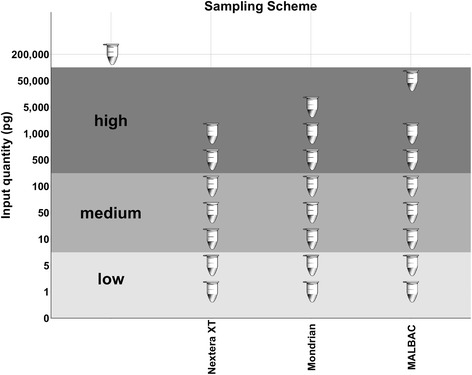
Sample overview. Each tube on this plot represents a mock metagenomic library preparation. The control library is an unamplified TruSeq library of the same mock community sample generated from 200 ng input DNA

For our defined mock community of 26 microorganisms encompassing 23 bacteria and 3 archaea, we generated and sequenced Illumina libraries using Nextera XT, Mondrian and MALBAC protocols for serial template dilutions ranging from 50 ng - 1 pg (Fig. 1). Following read trimming and quality filtering, all datasets were randomly subsampled to 15 million reads and all downstream analyses were performed on the subsampled data. Total read counts and the percentage of reads following retained trimming and quality filtering are reported in Additional file 1: Table S2.

The percentage of reads lost to trimming and quality filtering was fairly consistent across library types and input quantities, where 11 % of reads were removed from the control library and a similar fraction was removed from the Mondrian, MALBAC and high input Nextera XT libraries. However, with decreasing input levels in the Nextera XT libraries, the fraction of reads removed increased (Additional file 1: Table S2), which is likely a result of the increased activity of the transposase mediated fragmentation reaction at the lowest inputs. The percentage of reads retained following read QC differed between library types (Overall ANOVA *p*-val < 0.001) and this difference appears to be driven by the percentage of reads removed from the Nextera XT libraries as compared to the Mondrian and MALBAC libraries (Additional file 1: Table S2). In addition to the percentage of reads retained following trimming and quality filtering, the percentage of duplicate reads also differed across library types (Overall ANOVA *p*-val < 0.001). This was especially noticeable in the Nextera XT libraries (Additional file 1: Table S2), although all libraries generated a fairly large fraction of duplicate reads, which may be related to the low diversity of our mock community.

### Insert size variability

**Fig. 2:**
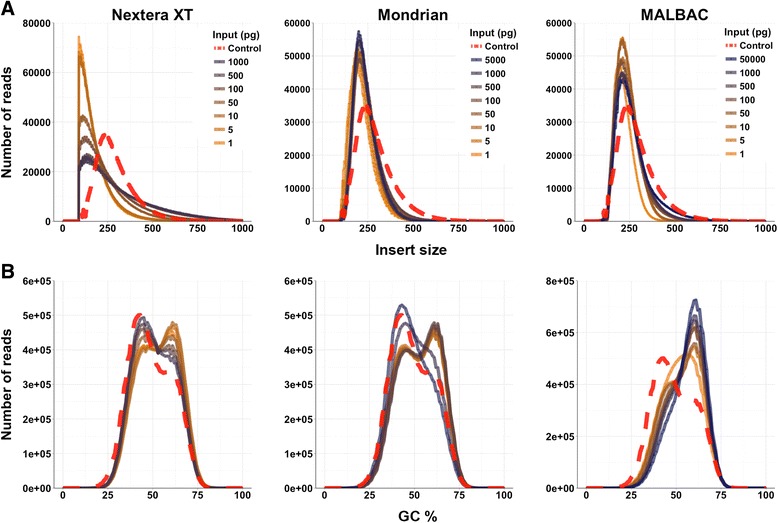
**a** Insert size and (**b**) GC profiles of Illumina sequence data from each of the three different library preparation methods. Unamplified control library is represented by the red dashed line

Library preparation had a strong effect on library insert sizes (ANOVA, *p* < 0.001) (Fig. 2a and Additional file 1: Table S3). The mean insert size of the control TruSeq library was 237 bp, which approximates the desired insert size as the TruSeq library contained sufficient starting material (200 ng) and no PCR enrichment cycles. The mean insert size for the Nextera XT, Mondrian and MALBAC libraries were 110, 200 and 208 bp, respectively. Conversely, input level did not have an effect on insert size distributions (high, medium and low) (ANOVA, *p* > 0.05) (Additional file 1: Table S3). The largest differences in insert size distributions occur between libraries originating from Covaris sheared input DNA and libraries prepared by the Nextera XT tagmentation procedure. The effect of library type on insert size was evaluated further using pairwise comparisons and Bonferroni adjusted *p*-values indicating that the Nextera XT peak insert sizes significantly differed from both Mondrian and MALBAC insert sizes (*p* < 0.001 in both cases) while the Mondrian and MALBAC insert sizes were not statistically distinct (*p* > 0.05) (Fig. 2a and Additional file 1: Table S3). These results are expected given that enzymatic shearing is generally more biased with regard to DNA fragmentation, as compared to mechanical methods, such as sonication [17].

### GC shifts with decreasing input levels

**Fig. 3:**
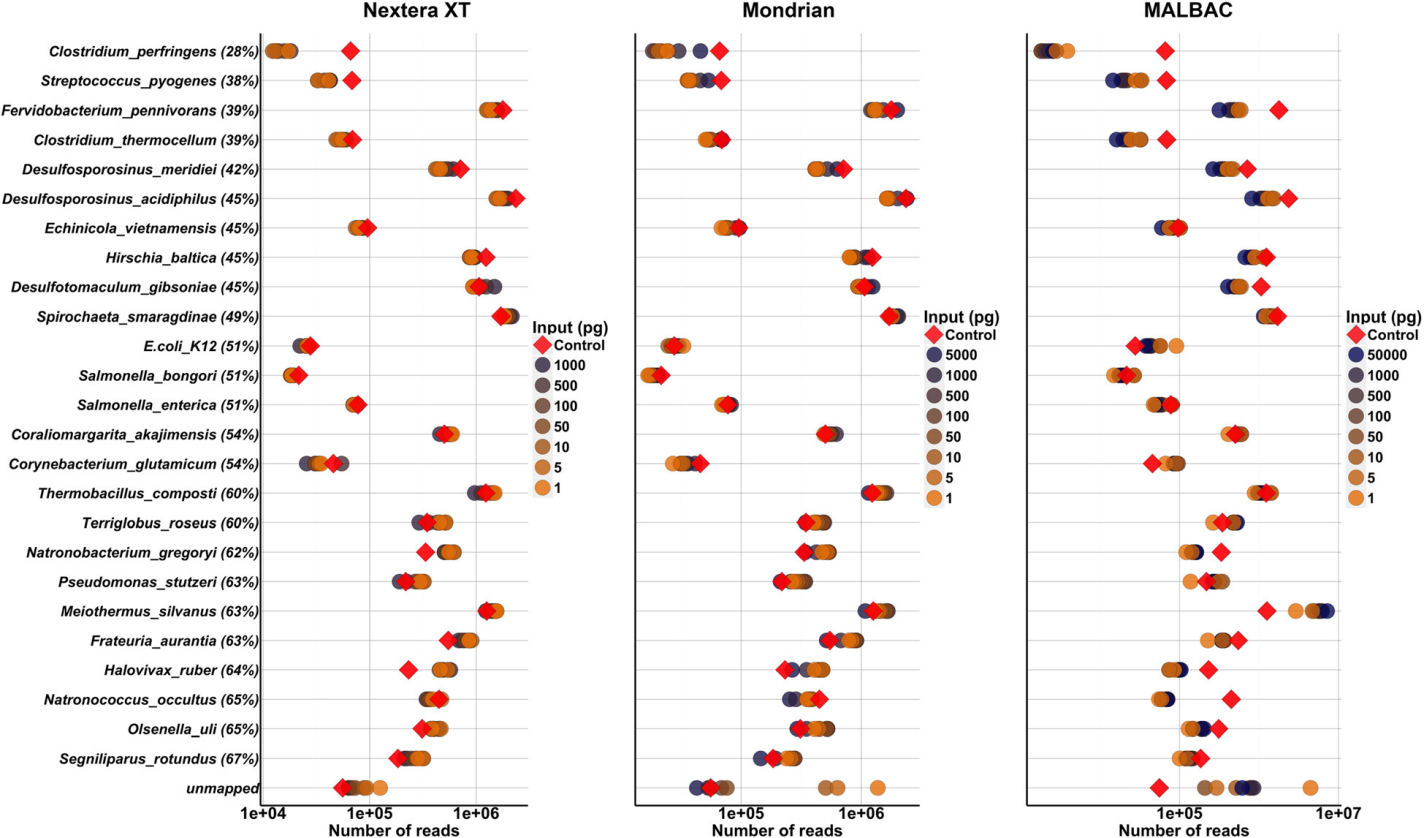
Relative abundances of each mock community member and the unmapped reads with the exception of *Nocardiopsis dassonvillei* (too few sequences mapped to reference). Individual GC plots corresponding to the high and low GC references are displayed in Additional file 1: Figure S1, which further illustrates the shift in relative abundance from low to high GC organisms across the dilution series. Unamplified control library is represented by the red diamond

GC profiles showed minor differences between the three library types, however shifts were apparent within the Nextera XT and Mondrian dilution series (Fig. 2b). As input level decreased, a shift toward a more GC rich community was observed. The GC profile of the unamplified TruSeq control contained a dominant GC peak at 43 % and a minor peak at 58 %. The MALBAC libraries displayed a very different profile than the control, while Nextera XT and Mondrian libraries gradually shifted to GC rich profiles where the libraries with the highest GC content correspond to the lowest input quantities (Fig. 2b). To analyze these profiles statistically, we performed a simple ANOVA on the dominant GC peak (GC percentage) containing the highest number of reads (dominant peak in Fig. 2b) across the three library types and the three input levels (low, medium and high). Library type had no statistical effect on GC profiles (ANOVA, *p* > 0.05), while input level did exhibit an effect (ANOVA, p = 0.05) (Additional file 1: Table S3). Following a Bonferroni *p*-value adjustment for multiple tests, no significant pairwise comparisons were observed, although the high versus low and high versus medium comparisons (Fig. 2b) suggest the potential for shifting GC profiles from low to high GC %, reflecting the trend that the high input libraries were more similar to the control than the lowest input libraries. This pattern is likely the result of an increase in PCR enrichment cycling just prior to flow cell loading and sequencing. To analyze this effect in more detail, we plotted the number of reads that mapped to each reference organism, and arranged the reference taxa from low to high GC %, which enabled the determination of the taxa driving the shift in overall metagenome GC profiles. The abundances of low and high GC organisms changed through the dilution series (Fig. 3), where a gradual drop in abundance was observed in the following low GC organisms: *Clostridium* perfringens, *Streptococcus* pyogenes, *Ferividobacterium pennivorans* and *Clostridium thermocellum* with a concomitant increase in the high GC organism abundances including *Halovivax ruber*, *Olsenella* uli and *Segniliparus rotundus.* Furthermore, the GC profiles of all reads that failed to map to the references (unmapped reads) were also skewed toward high GC content (Additional file 1: Figure S1). Together, this suggests a shift in GC content and a corresponding shift in mock taxa abundances that will eventually lead to artificial shifts in overall metagenome composition.

### Read mapping to reference genomes reveals differences between library preparations and input levels

**Fig. 4:**
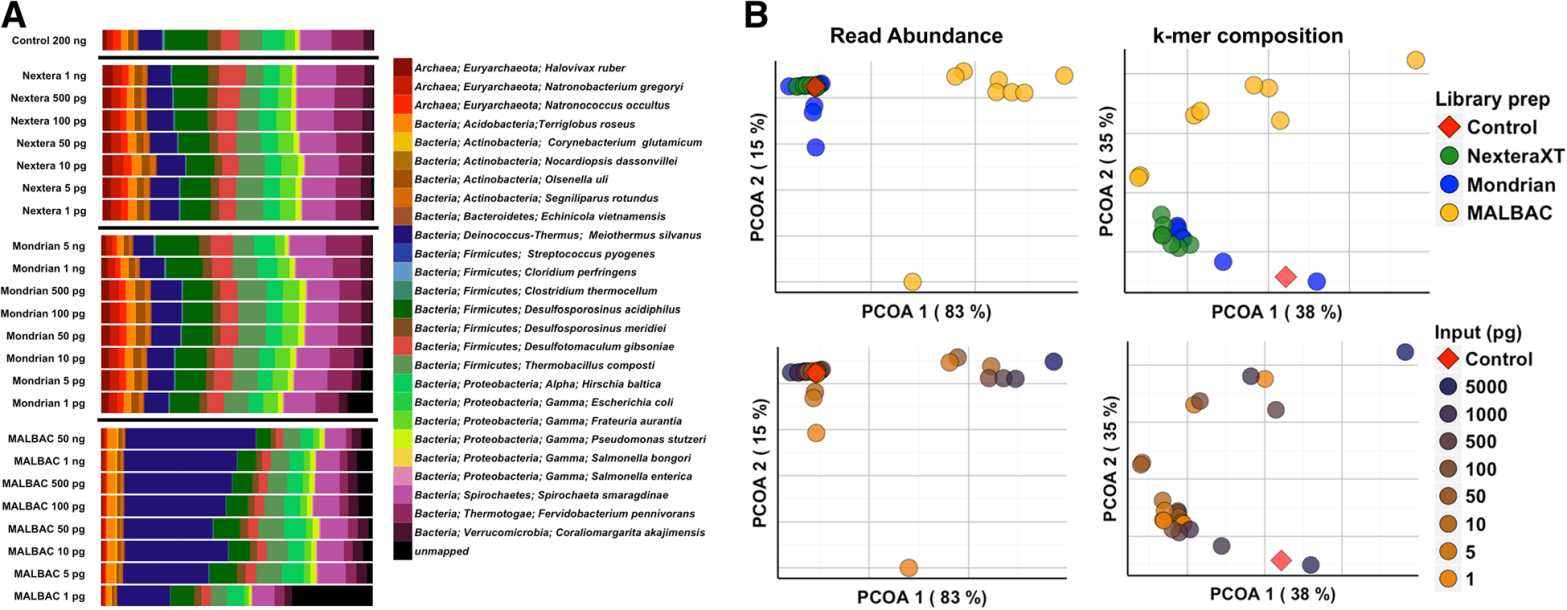
**a** Mock community relative abundances across each library prep kit and across each dilution including the TruSeq 200 ng Control library (***top***). **b** Principal coordinates analyses of Euclidean distances derived from mapping reads to the mock community reference genomes (***left column***) and of k-mer frequencies (***right column***, averaged sample k-mer frequencies from k2-k10 sample by sample k-mer distance matrices). Individual samples are colored by either library preparation (***top***) or starting input quantity (***bottom***). The unamplified 200 ng control library is represented by the red point in each ordination

Compared to the unamplified TruSeq control, the Nextera XT and most Mondrian libraries (top 6 dilutions, 5 ng – 50 pg) produced very few (less than 1 %) unmapped sequence reads (Additional file 1: Table S2, Figure S1B) and the taxonomic distributions were similar to the unamplified control library (Fig. 4a), although as stated above, taxa abundances did change with decreasing input levels. In contrast, the MALBAC libraries differed from the unamplified control throughout the dilution series. For example, *Meiothermus silvanus* was consistently enriched in the MALBAC libraries, while the relative abundances of *Natronobacterium gregoryi, Olsenella uli, Segniliparus rotundus, Halovivax ruber* and *Desulfotomaculum gibsoniae* decreased (Fig. 4a) when compared to the control library. The number of unmapped reads in the MALBAC libraries was also relatively high, above 1 % across all libraries and nearly 30 % in the 1 pg library. To further characterize the unmapped reads, the unmapped sequences were reclassified with FOCUS, a short read classifier based on a database of 2766 reference genomes [18]. This allowed us to determine if reads were simply below the quality required for mapping or if they represent common contaminants that overwhelm the target DNA during library preparation. The unmapped reads identified in the MALBAC libraries represented a relatively large proportion of total reads (Fig. 4a and Additional file 1: Table S1), and many of these reads were surprisingly reclassified to *Meiothermus silvanus*, a member of the mock community, which implies that these reads were below the quality required for the initial reference based read mapping. The remaining unmapped reads from the MALBAC libraries were reclassified with FOCUS to a variety of taxa not included in the mock community (Additional file 1: Figure S2), which likely represent reagent and/or lab contaminants that become more pronounced at the lowest dilutions, which is one of the inherent risks when working with low-biomass samples. By contrast, the majority of the unmapped reads from the 5 and 1 pg Mondrian libraries (Additional file 1: Figure S2) could not be assigned to the FOCUS database with the exception of some *Mycoplasma pneumoniae* assignments, indicating low quality reads at the lowest inputs (Additional file 1: Figure S2).

To further explore whole library differences where each sample represents an individual microbial community, we computed Euclidean distances of all pairwise library comparisons, and performed principal coordinates analyses (PCoA) on these distances to determine whether library type and/or input level had an effect on the overall composition of the mock metagenomes. Based on the Euclidean distance PCoA plot in Fig. 4b (left column), the Nextera XT and Mondrian libraries appear highly similar to the control library while the MALBAC libraries formed their own distinct cluster (Fig. 4b left column). These patterns were supported using PERMANOVA statistics on the distance matrices with library type as the grouping factor, excluding the control sample as n = 1 (PERMANOVA *p* < 0.001, Additional file 1: Table S4). Both Nextera XT and Mondrian libraries significantly differed from the MALBAC libraries (Nextera XT vs MALBAC: *p* = 0.006, and Mondrian vs MALBAC: *p* = 0.006, Additional file 1: Table S4). When libraries were grouped by input level (high, medium and low), no effect was observed (PERMANOVA *p* > 0.05, Additional file 1: Table S4), however there does appear to be a within library gradient where libraries of higher input are more similar to each other than they are to the lower input libraries (PCoA Fig. 4b, left bottom plot).

### Sequence signatures demonstrate compositional differences between library types

To supplement the community comparisons using read abundances, we also used k-tuple frequencies to determine the effect of library type and input level on the resulting metagenomic composition irrespective of reference database. This approach calculates k-mer frequencies across a range of k-mers (k2-k10), then generates distance matrices used as input for ordinations and multivariate statistical analyses [19]. The results indicate similar patterns to the read mapping results, as library type (PERMANOVA *p* = 0.001, Fig. 4b right column, Additional file 1: Table S4), but not input level (PERMANOVA *p* > 0.05, Fig. 4b right column, Additional file 1: Table S4) significantly influenced the k-tuple frequency of the mock metagenomic libraries. However, there again appears to be a slight gradient where the higher input Mondrian and Nextera XT libraries cluster closer to the control than the lower input libraries (Fig. 4b right bottom plot). We justified the grouping of different k-mer length profiles by performing a Procrustes analysis on small (k2-k3), medium (k4-k6) and large (k7-k10) k-mer length profiles in order to ensure reproducibility of community profiles at different k-mer lengths. As observed in Additional file 1: Figure S3, the effect that library type had on metagenomic sequence composition is present regardless of k-mer length.

### Assembly quality varies across library type and input level

*De novo* assemblies were generated for each sample and the associated assembly statistics are presented in Additional file 1: Table S2 and Table S5. The control unamplified TruSeq library had an overall assembly size of 60 Mb, total number of contigs was 43,848, contig N50 was 1271 bp, largest contig was 1.3 Mb and 95 % of reads could be mapped back to the assembly. Overall, the assembly stats of the three tested library preparations were variable (Additional file 1: Table S2 and Table S5), although there are a few consistent patterns worth noting. The percentage of reads that mapped back to the corresponding assemblies gradually declined with decreasing DNA input. Moreover, the largest contigs for each of the tested library types were considerably smaller than the largest contig generated from the unamplified control library.

### Reference independent binning produces near complete genomes of many of the dominant mock community microbes

**Fig. 5:**
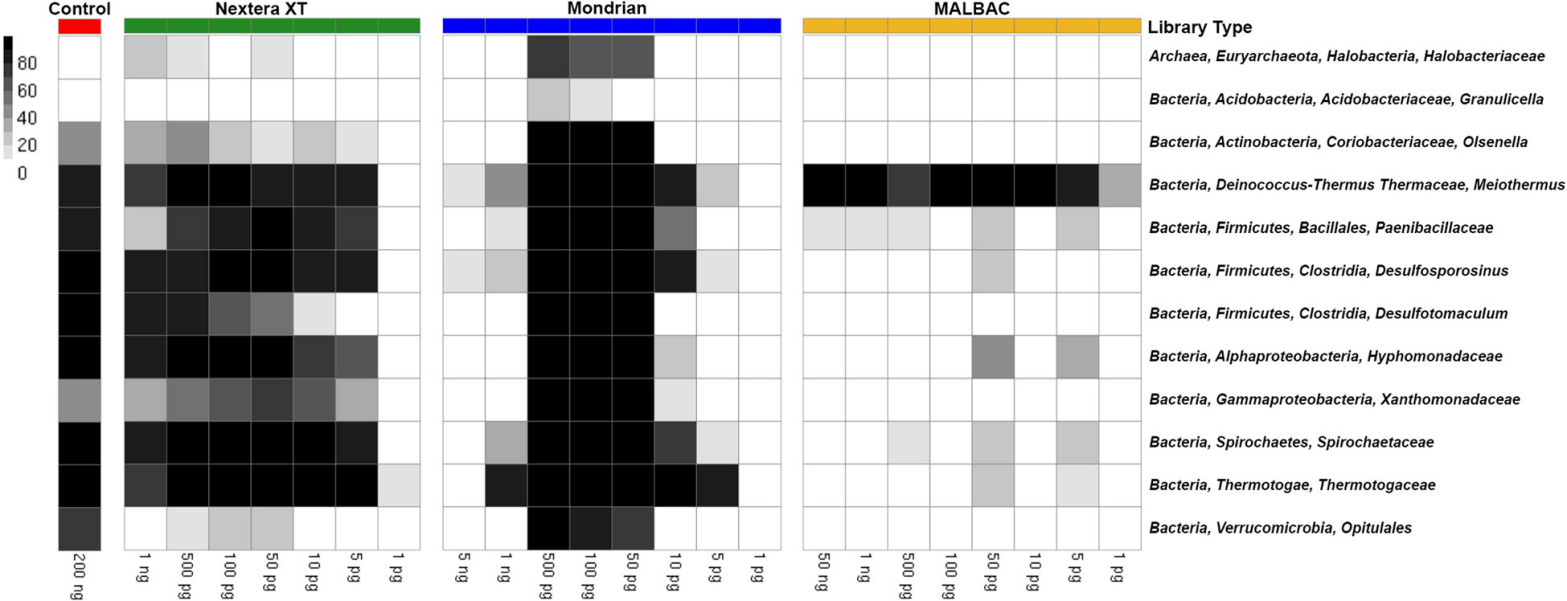
Heatmap noting the completeness of genomic bins extracted from each of the low input metagenomes. The color bar on top of the figure refers to each of the three tested library types and control library (Control = red, Nextera XT = green, Mondrian = blue and MALBAC = yellow). Samples are also arranged where the highest input quantity is located on the left side of each library type

To complete our current analyses, we binned genomes from each of the low input metagenomic libraries and compared the distribution and completeness (based on the presence of co-located marker gene sets) of the extracted bins to the bins extracted from the unamplified control library. The employed binning tool, MetaBAT, uses a reference independent approach to bin genomes based on coverage and tetra-nucleotide frequency [20]. Provided that our low input library comparisons required subsampling to allow sample to sample comparisons, our intent was not to assemble all the genomes present in the mock community, but instead to make meaningful comparisons between the bins extracted from the ideal unamplified control library to the low input libraries. Based on the binning results and bin quality assessments, ten near complete, taxonomically distinct genome bins were extracted from the control library. A very similar genome bin profile was observed in the Nextera XT libraries down to 5 pg, however no bins could be extracted from the 1 pg library (Fig. 5). Interestingly, the Mondrian libraries produced high quality bins in the middle of the dilution series while few bins could be extracted from the highest and lowest input samples. This observation appears to mimic the assembly statistics derived from the Mondrian libraries, where the best assemblies were obtained from the libraries between 500 and 50 pg input material. Consistent with the read and composition based analyses described above, the MALBAC libraries were the most biased of the three tested protocols. Only one taxonomic bin could be successfully extracted (Fig. 5), a *Meiothermus silvanus* bin, which is expected based on the over-representation of this mock community member in the MALBAC libraries (Fig. 4a).

## Discussion

Based on insert sizes, GC content, read mapping, k-mer frequency and assembly statistics, the low biomass libraries with the highest similarity to the 200 ng unamplified control were the Nextera XT libraries followed by the Mondrian libraries, and finally, the MALBAC libraries. While no library type achieved best results across all metrics used in our current study, the Nextera XT libraries performed topmost, as the only quality metrics significantly departing from the control were peak insert size, GC content at the lowest inputs, and some of the assembly statistics.

### Tagmentation produces variable insert sizes

Enzymatic fragmentation based on the Tn5 transposase has been described as a highly efficient library preparation method [21], with the main obstacle being the control of library insert size. The Nextera XT transposase produced insert sizes that were on average considerably smaller than the other library types (Fig. 2a), which is consistent with the observations noted by Chafee et al. [16] and Adey et al. [17], suggesting increased activity and the potential for slight sequence dependent biases at the lowest template levels. Few studies have analyzed the effect of over fragmentation in Nextera XT libraries, however work by Marine et al. found that even the smallest inserts (<100 bp) recruited to viral reference genomes at a similar frequency to the longer sequences used in their study (>500 bp) [22]. This is similar to our own read mapping results, as a high fraction of reads were mapped back to the respective assemblies throughout most of the dilution series with the exception of the lowest input, 1 pg library (Additional file 1: Table S2). However, paired-end 150 bp reads may result in truncated reads when inserts are < 150 bp, and redundant sequence data when inserts are < 300 bp; reduced efficiency of small inserts may explain the slightly poorer assembly results from the Nextera XT data.

### Variation in library GC content may be related to increased PCR cycling at the lowest inputs

While PCR enrichment is an often necessary step in the production of sufficient adapter ligated library molecules for sequencing, this step can lead to an artificial, albeit stochastic shift in GC content [23, 24]. For example, Aird et al. examined sources of potential bias through the course of an Illumina library preparation including mechanical shearing, ligation of adapters and the PCR enrichment step. They noted that PCR led to the largest bias, as the coverage of both AT and GC rich portions of the *E. coli* K12 genome dropped dramatically following as few as 10 PCR cycles [25]. Interestingly, the three tested library types in our current study each had a significant GC shift below 500 pg input DNA (Fig. 2b). Similarly, Chafee et al. [16] observed a GC shift in *Arabidopsis* associated metagenomic communities with decreasing input. While the direction of the GC shift observed by Chafee et al. was the opposite direction of our current analyses (AT rich fragments increased in *Arabidopsis* community while GC-rich fragments increased in our current work), this shift may be organism specific [14, 26], and is therefore difficult to generalize across whole microbial communities. What can be generalized is that some form of PCR amplification bias is occurring, which leads to slightly altered abundance profiles at the lowest input levels (Fig. 4 and Additional file 1: Figure S1). Consequently, the number of PCR cycles used prior to and following adapter ligation should be kept to a minimum to avoid GC-based shifting community composition, which would make quantitative analysis prohibitive.

### Taxonomic assignment of reads to references is minimally biased in the Nextera XT and Mondrian libraries down to picogram levels

Since this work was performed on a defined mock community, our analyses represent a systematic overview of the factors with the greatest impact on the subsequent metagenomic sequence data. The mapping of reads to references demonstrated that the Nextera XT libraries were most similar to the unamplified control library with the fewest unmapped reads throughout much of the dilution series (Fig. 4). The number of unmapped reads observed in the Mondrian samples exceeded 1 % in the 5 and 1 pg libraries, while all MALBAC libraries contained high levels of reads that could not be mapped back to the reference genomes, suggestive of significant biases associated with the MALBAC amplification procedure. The MALBAC procedure is intended to reduce bias by suppressing the over-amplification of abundant template molecules, has recently been shown to produce unbiased coverage across human cancer cell lines [15] and has performed as well as other single-cell amplification methods such as MDA on single-cell templates [27]. However, both studies examined the value of the MALBAC procedure on isolated single cells, not mixed metagenomic populations. Based on our current work, this method clearly over-amplifies some taxa at the expense of others, making it unsuitable for use on low biomass environmental samples (Fig. 4).

The rather small increase in the number of unmapped reads at the lowest Nextera XT inputs and the slightly larger increase in unmapped reads in the 5 and 1 pg Mondrian libraries suggests that low input libraries using either the Nextera XT or Mondrian systems are suitable for picogram range DNA samples with the understanding that some biases may occur as the number of PCR enrichment cycles is increased. However, as other recent microbiome studies have pointed out, contaminants become increasingly important and problematic at low target DNA quantities [28–31]. Therefore, both wetlab scientists and bioinformatic analysts need to be aware of the effect of additional PCR cycling on overall community composition, and the increasing influence of contaminants with low amounts of starting material.

### Comparative metagenomics using community based analyses

In addition to the general library statistics and taxonomic read mapping, we also took a community ecology approach to assess the variability between library types and input quantities. Based on these analyses, we found that library type had a significant effect on metagenome composition (both read mapping and k-mer analyses). While input level had no significant effect, a slight gradient separating the high and low input levels in the k-mer based principal coordinates plots was apparent (Fig. 4b). Although Chafee et al. used a slightly different experimental approach, they too did not observe a significant effect of either input level or PCR cycle number on Nextera XT metagenomes [16]. In our current work, we could not decouple the effect of PCR cycle number and input quantity, as an increase in PCR cycles was needed for library production from the lowest inputs. The MALBAC procedure clearly generated the most distinct libraries of the three tested protocols, and these libraries were also clearly different from the unamplified control library (Fig. 4b and Additional file 1: Figure S3). While this technology has been previously shown to produce similar results to other single-cell amplification methods [27], our results suggest that this method is not well suited for low biomass metagenome studies (Fig. 4).

### Assembly quality varies across library types with potential impact on downstream analyses

Assembling reads into larger contiguous fragments is becoming increasingly important in metagenomic studies. Read lengths will continue to increase and assembly algorithms specific for metagenomes are now being developed [32–34]. In our current study, the assembly statistics for both Nextera XT and Mondrian libraries were acceptable, however neither library produced the assembly quality of the unamplified 200 ng control library (Additional file 1: Table S2). To further assess assembly quality, we binned the contigs from each library into distinct genomic bins and determined the corresponding bin completeness using sets of lineage specific co-located marker genes [35]. Ten taxonomically distinct bins were created from the 200 ng unamplified control library, which was mirrored in the Nextera XT libraries with the exception of the 1 pg library. The same genome bins were extracted from the middle input (500 – 50 pg) Mondrian libraries (Fig. 5). The drop off in binning efficiency at the lowest input levels is likely the result of decreased assembly quality at the lowest inputs as noted in Additional file 1: Table S2. In contrast, the MALBAC libraries only produced a single *Meiothermus silvanus* bin throughout the dilution series, which likely reflects the compositional bias associated with this library preparation procedure (Fig. 4a). Overall, our current work suggests that satisfactory assembly quality can be achieved using the Nextera XT kit down to 5 picograms of input DNA with little effect on downstream analyses such as extracting genomes from metagenomes (Fig. 5). High quality assemblies are often a prerequisite for meaningful functional annotation, as annotation based on either short reads or many short contigs will likely result in fragmented gene predictions or may fail proper annotation altogether. Therefore, based on our current work, the Nextera XT protocol produces high quality, low input metagenomic libraries at extremely low inputs suitable for a variety of downstream analyses.

## Conclusions

The motivation behind our current work was to determine the lower limits of metagenomic library preparation protocols and to assess which library prep performed best down to single picogram DNA input levels. We show that despite the typical biases associated with the PCR enrichment step, high quality metagenomic libraries can be produced at low picogram levels, although more DNA is desirable to minimize the risk of contamination and maximize the read and assembly quality metrics as discussed. Of the three tested library preparation protocols, the Nextera XT and Mondrian protocols produced the highest quality libraries across all dilutions. From a production standpoint, the Nextera XT library preparation kit is most amenable to a high throughput workflow, as it is a quick library preparation procedure that can be performed in 96-well format with limited hands-on time. This study lays the groundwork for the continued metagenomic exploration of low biomass ecosystems without the use of pre-enrichment steps such as MDA. Lastly, as the field of microbial genomics is moving towards the isolation and sequencing of microbial aggregates, microcolonies and/or single cells where MDA has been a requirement to date, we are gradually transitioning to a new sequencing era where the output of As, Ts, Gs and Cs will no longer be limited by the amount of starting material.

## Methods

### Description of mock community composition

The mock community is composed of 23 bacterial species and 3 archaeal species (Additional file 1: Table S1). DNA from pure cultures of each of the 26 microbial taxa was extracted with standard genomic purification kits. DNA extracts were quantified in quadruplicate with the Qubit 2.0 fluorometer and pooled at varying ratios to produce a mock community representative of a low diversity metagenome sample (Additional file 1: Table S1).

### Library preparation and sequencing

DNA from the same pooled mock community sample was used as input for each of the different library preparation procedures. The unamplified control library was prepared from a 200 ng aliquot of the pooled mock community DNA using Illumina’s TruSeq library preparation protocol. Ten-fold dilutions of extracted genomic DNA were prepared for Nextera XT, Mondrian and MALBAC libraries. The dilution series used in Mondrian and MALBAC library preparation and the 200 ng control aliquot were each subject to mechanical shearing using the Covaris Adaptive Focused Acoustics instrument resulting in 300 bp fragments. Following fragmentation, TruSeq, Mondrian and MALBAC libraries were prepared following manufacturers instructions. Briefly, the Mondrian libraries were prepared using the Mondrian SP+ microfluidics system and NuGen’s Ovation Ultralow DR Muliplex kit using 9 PCR enrichment cycles for the 5 ng sample, 15 cycles for the 1 ng sample and 20 cycles for all samples below 1 ng. MALBAC libraries were prepared using 8 linear pre-amplification rounds using MALBAC specific primers, which enables looping of full amplicons, preventing the overamplifcation of high abundance fragments. Following the MALBAC pre-amplification rounds, an additional 12 cycles were performed on samples ≥ 1 ng, 13 cycles for samples between 500 pg and 50 pg and 14 cycles for samples ≤ 10 pg. Finally, the tagmentation based Nextera XT libraries were prepared following the Nextera XT protocol with 12 PCR enrichment cycles for the inputs from 1 ng – 100 pg and 15 PCR enrichment cycles for the 50 – 1 pg Nextera XT libraries. All libraries were sequenced on the Illumina HiSeq 2000 platform using 2 x 150 bp paired end sequencing [36]. The dilutions and overall sampling scheme used are illustrated in Fig. 1.

### Library quality control, trimming, read mapping, de novo assembly and binning

All libraries were quality checked and trimmed in the same manner. Briefly, quality trimming, contaminant removal and adapter trimming were performed using the bbtools (http://sourceforge.net/projects/bbtools/) module bbqc.sh. The percentage of duplicate sequences was calculated using the bbtools dedupe.sh module. Duplicated reads were reported but not removed from downstream analyses, as it remains difficult to designate a duplicated sequence as either artificial or natural, especially in a low diversity community [16] such as this 26 member mock community used in our current study.

Quality checked, trimmed reads were subsampled to 15 million reads per sample to allow library to library comparisons, both at the read and assembly levels. Following subsampling, reads were mapped back to the 26 reference genomes using the module bbsplit from the bbtools package (http://sourceforge.net/projects/bbtools/), which allows mapping of reads to multiple references simultaneously. Reads were assigned as unmapped if they did not map to one of the reference genomes at 95 % similarity or higher. If reads map to multiple references, reads are assigned to the reference that they map to best (highest % similarity). Following read mapping, a taxa by observation (sample) table was made in order to compare libraries and perform the relevant statistics.

The quality checked, trimmed and subsampled reads were used as input for our in-house metagenomic assembly pipeline. This pipeline uses SOAPdenovo [37], a short read assembler that uses six different k-mer lengths (soap81, soap85, soap89, soap, 95, soap87 and soap101). This pipeline follows recommendations laid out in Scholz et al. 2014 [38]. Briefly, different k-mer sizes were used as different assemblies can result from different k-mer lengths and smaller k-mer lengths can help assemble genomes present at lower abundance. While a higher number of missassemblies may occur in smaller k-mer length assemblies, it is believed that assemblies with varying k-mer lengths will produce more complete assemblies [38]. Contigs generated from each assembly (6 total contig sets) were de-replicated, then sorted into two pools based on length. Contigs smaller than 1800 bp were reassembled using Newbler (Life Technologies, Carlsbad, CA version 2.8) in an attempt to generate longer contigs. All contigs larger than 1800 bp were then combined. Finally, reads were mapped back to the assembly using bbmap from the bbtools software package and assembly stats were generated using stats.sh, also from the bbtools software package (http://sourceforge.net/projects/bbtools/). Following assembly, we performed metagenomic binning using MetaBAT, which uses both coverage and sequence composition for the identification of bins from complex communities [20]. To estimate completeness of each genome bin, we used CheckM, a software package developed to assess overall bin quality based on co-located sets of lineage specific marker genes [35].

### Analysis of communities using k-tuple frequencies

Libraries were also compared using d2Tools, which is a sequence-signature based approach that counts the frequency of k-tuples (k2-k10) of each sample, then calculates pairwise dissimilarity matrices using various distance metrics [19]; Euclidean distances are reported here. Following distance matrix calculations, individual distance matrices for each k-mer depth were merged to a single distance matrix by calculating the mean distances across k-mer depths (k2-k10). To ensure that k-mer profiles of k2-k10 could be merged, k-mer profiles were split into short (k2-k3), medium (k4-k6) and high (k7-10) k-mer depths and the corresponding profiles were compared using Procrustes analyses (Additional file 1: Figure S3), which displayed similar profiles across the three binned k-mer depths.

### Statistical analyses

All statistical analyses and visualizations were performed with R version 3.1.1. The maximum insert size and GC profile peak heights that represent the dominant insert size and GC content were used as input for 1-way ANOVAs comparing both library type and input level (grouped into low, medium and high inputs, see Fig. 1). 1-way ANOVAs were also performed on the various assembly metrics used to assess the quality of the assemblies across library preparation protocols and input levels. Euclidean distance matrices and the corresponding principal coordinates were calculated on the taxa by sample table using Qiime [39] and plotted using R and the ggplot2 package. Euclidean distances generated by the d2-Tools k-mer profiling software were also used as input for principal coordinates calculations and corresponding visualizations using Qiime, R and ggplot2. Permutational multivariate analysis of variance (PERMANOVA) using all distance matrices was performed using the Adonis function from the R package vegan on the results of both the read (bbsplit) and k-mer based distance matrices (d2-Tools). For multiple comparisons such as all pairwise library type comparisons, *p*-values were adjusted using the Bonferroni correction.

## Availability of supporting data

The data sets supporting the results of this article are available for download at http://genome.jgi.doe.gov/LowBiomassRD/LowBiomassRD.info.html. For each library type: TruSeq Control, Nextera XT, Mondrian and MALBAC, there are raw fastq files and the corresponding assemblies.

## Additional file

Additional file 1:
**Supplementary Info.** (DOCX 3.22 MB)
