# Supplementary material for: Impact of library preparation protocols and template quantity on the metagenomic reconstruction of a mock microbial community
Source: BMC Genomics. 2015 Oct 24;16:856. doi: 10.1186/s12864-015-2063-6 (PMC4619416; doi:10.1186/s12864-015-2063-6)

**Supplementary Info**

**Supplementary Figures**


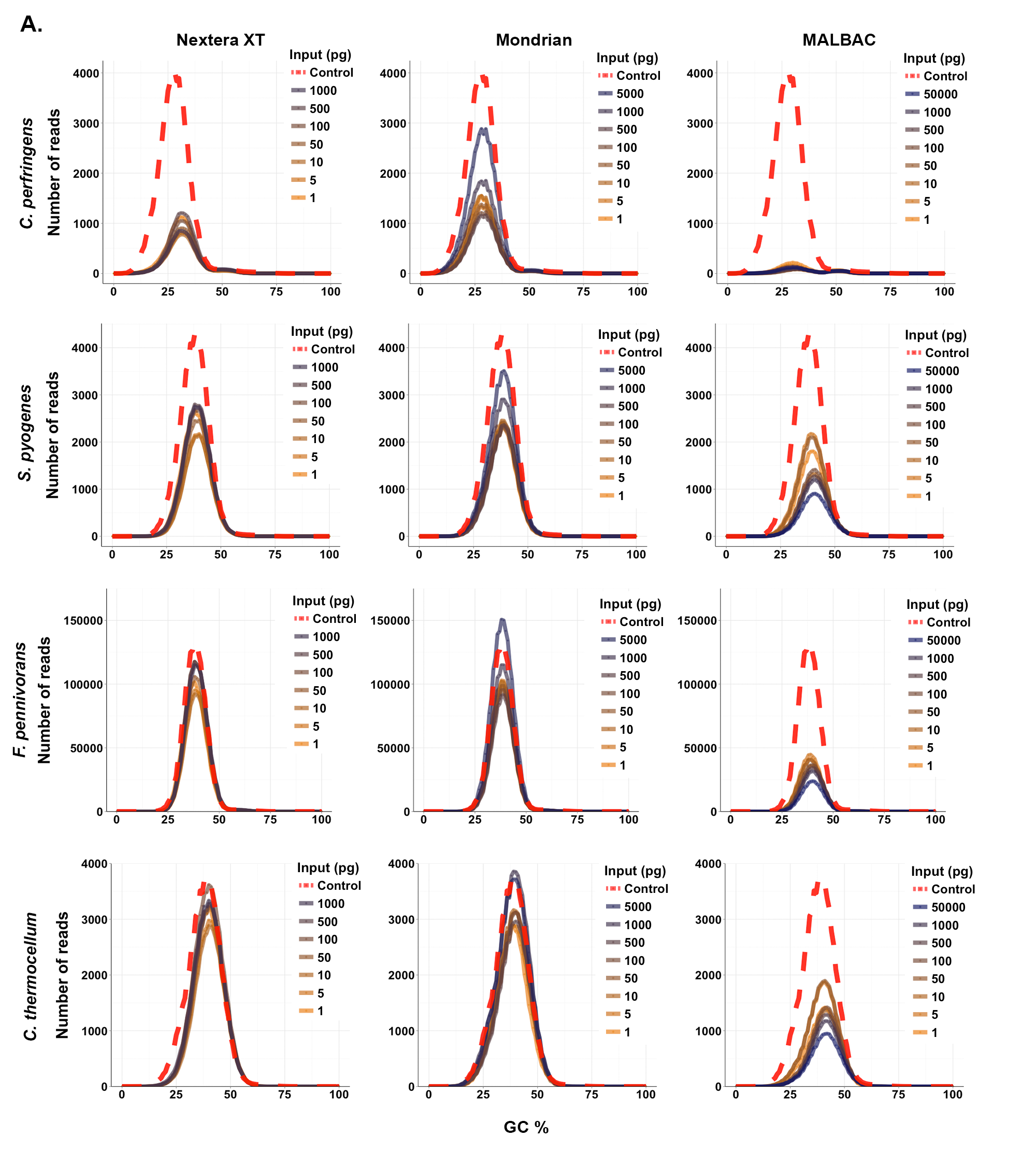


**
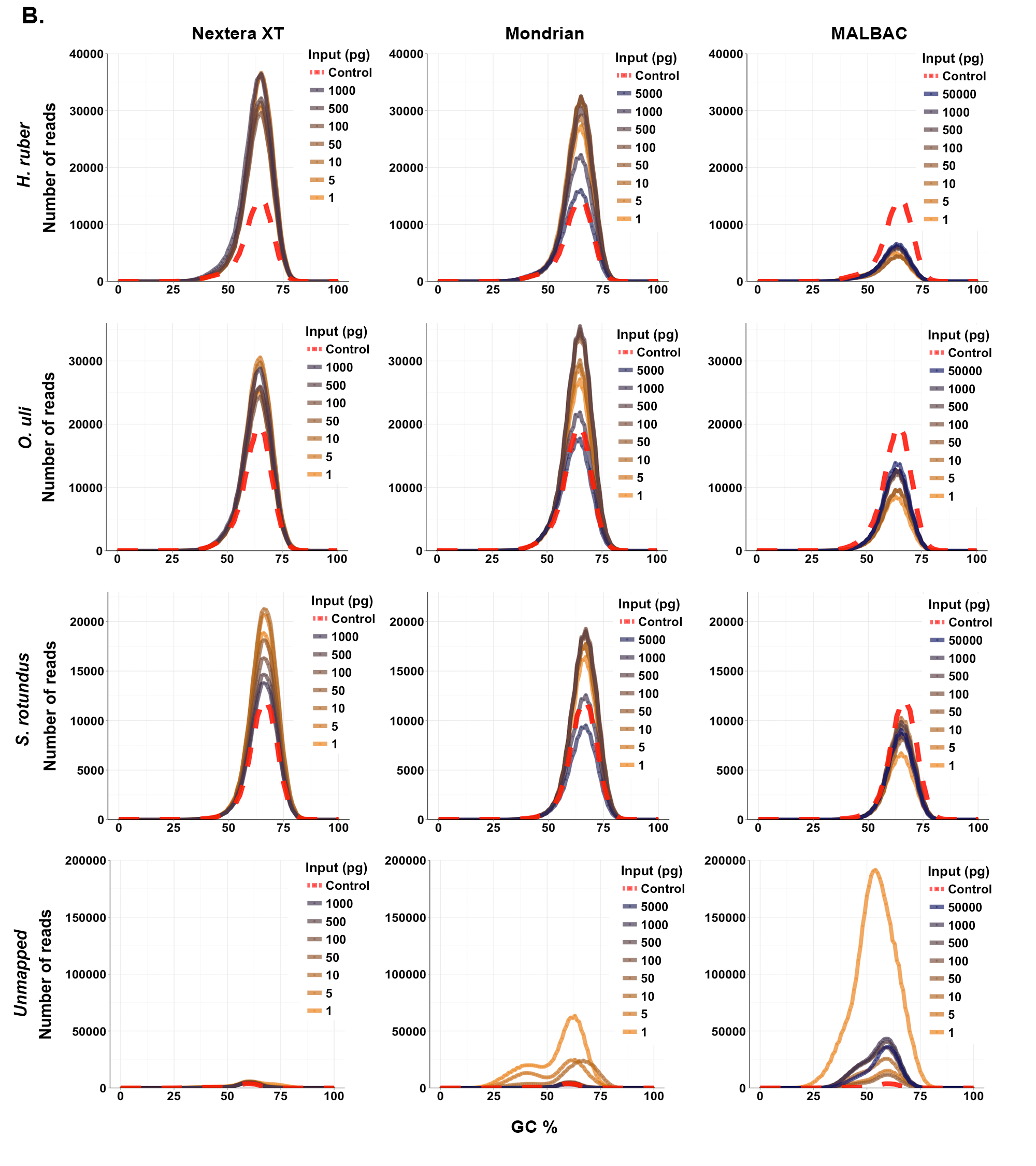
Figure S1.** GC profiles of the mapped reads that correspond to the 26 reference taxa. Low GC organisms are shown in panel (**A**) and high GC organisms in panel (**B**). Note the shift in abundance of each taxon at the lowest input levels. Unamplified control library is represented by the red dashed line.


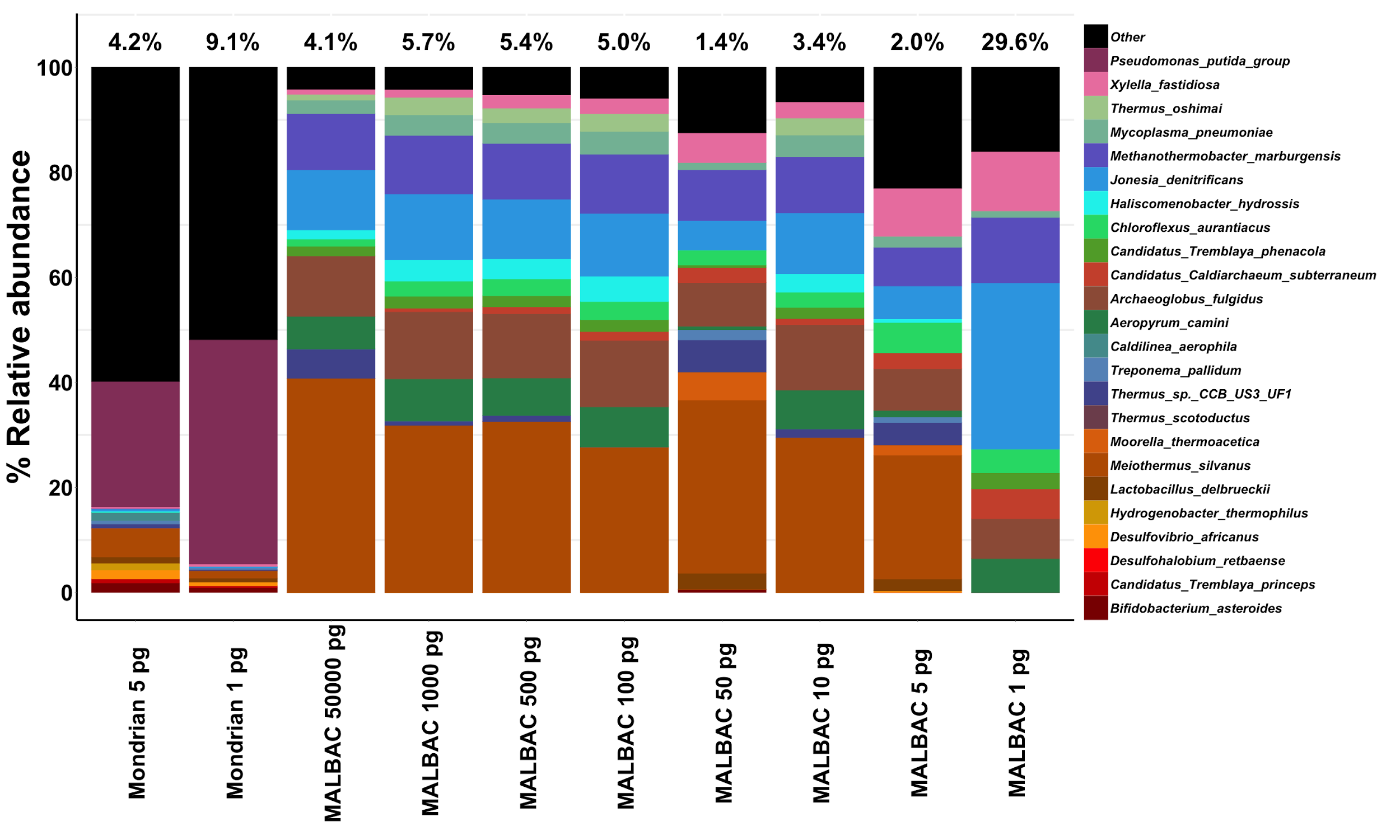


**Figure S2.** Additional classification of unmapped reads using FOCUS, which is an alignment-free/composition based taxonomy assigner that assigns taxonomy based on a reference composed of 2,766 high quality genome sequences. The percentage of the total number of unmapped reads per sample is shown above each bar.


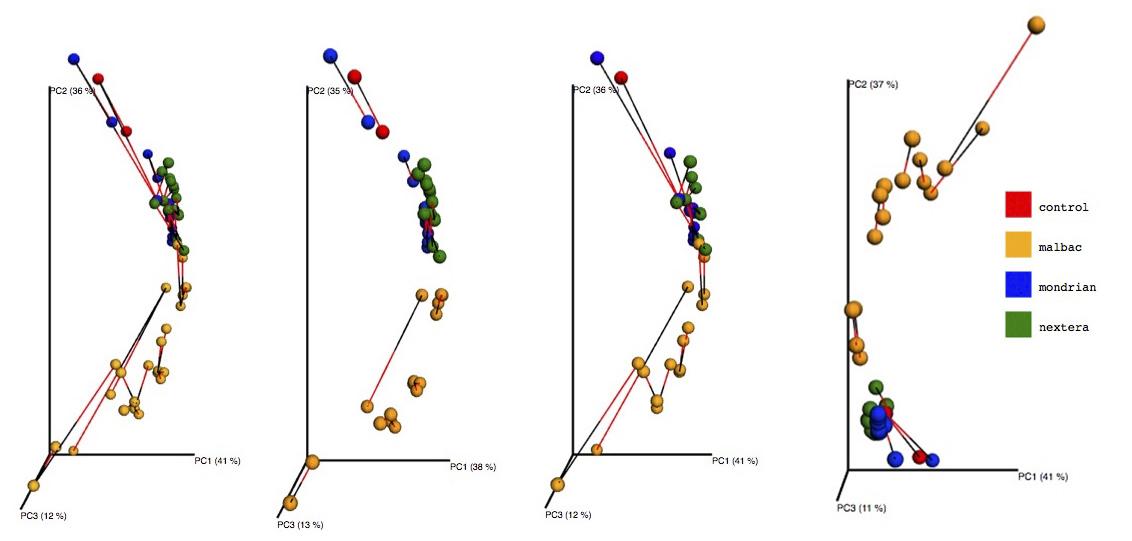


**Supplemental Figure 3.** Procrustes plots comparing (**A**) short, medium and high k-mer composition (**B**) short and medium k-mer composition, (**C**) short and high k-mer composition, and (**D**) medium and high k-mer composition. Lines between points represent paired samples**.**

**Table S1.** Mock community members alongside some basic genome characteristics including GC%, genome size and number of replicons. The input concentrations of each mock community member are also included. However these are only relative estimates based on Qubit fluorescence measurements and the ground truth for our current study is the comparison of all experimental libraries to the unamplified TruSeq control library.


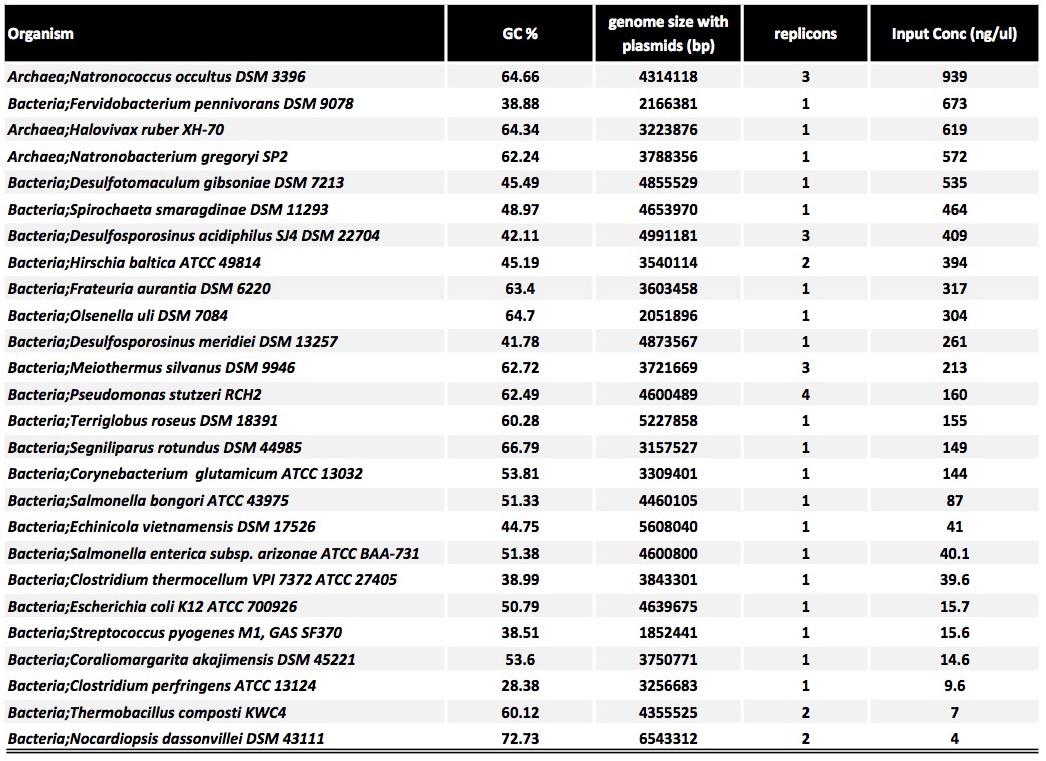


**Table S2.** Basic assembly and library stats including: total sequence count, the percentage of reads that remained following QC, the percentage of duplicates, the percentage of reads that could not be mapped to the reference genomes, total assembly size, # of contigs, contig N50, largest contig, and the percentage of reads that could be mapped back to the corresponding assembly


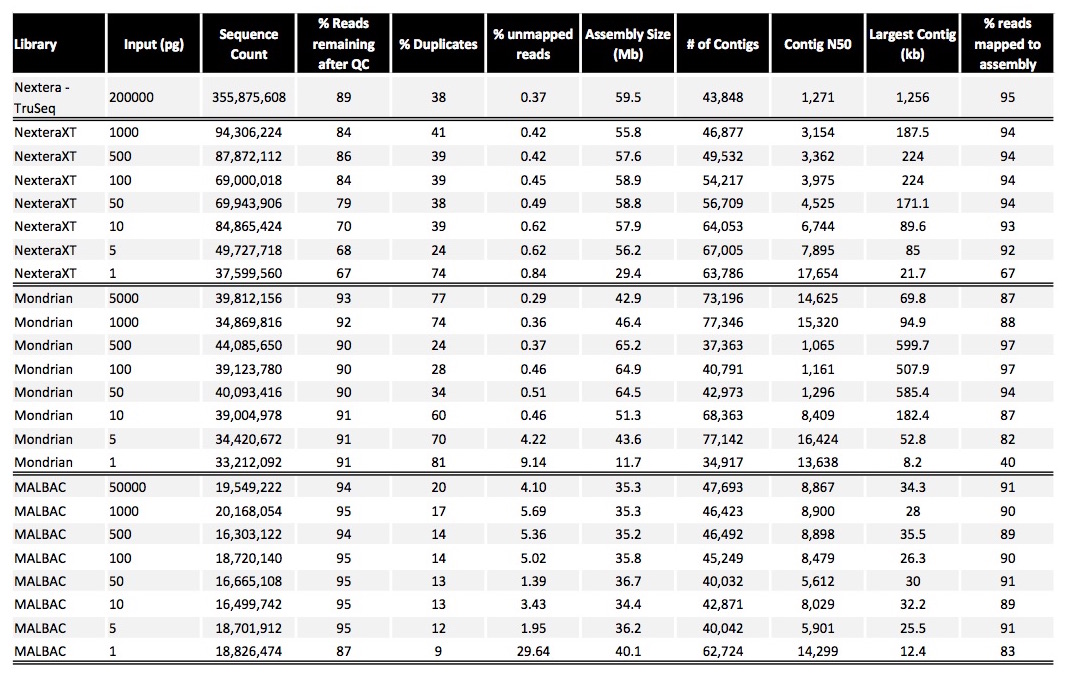


**Table S3.** ANOVA statistics on peak insert size and peak GC content across library type and input level. P-values resulting from multiple comparisons were adjusted using the Bonferroni correction.


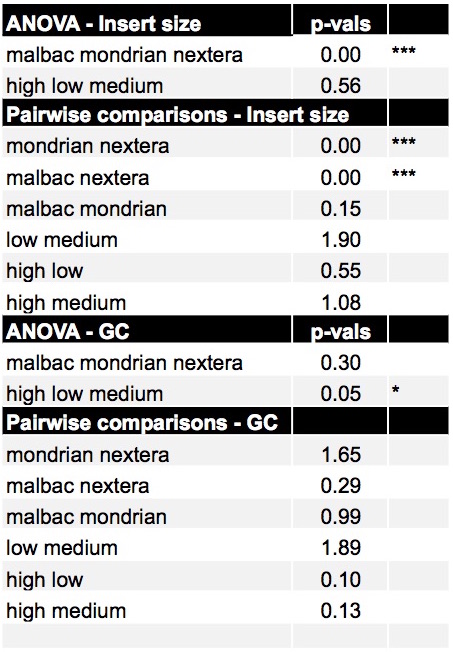


**Table S4.** Permanova on library type and input quantity comparisons using Euclidean distances between samples as input. P-values resulting from multiple comparisons were adjusted using the Bonferroni correction.
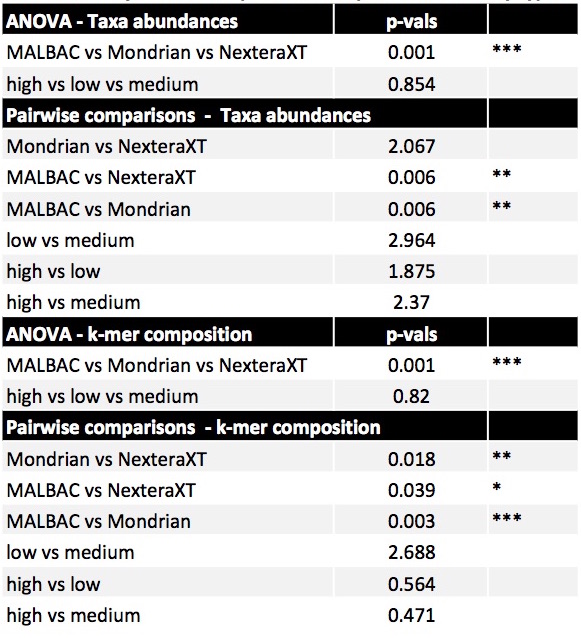


**Table S5.** ANOVA statistics on read and assembly metrics across library type and input level. P-values resulting from multiple comparisons were adjusted using the Bonferroni correction.


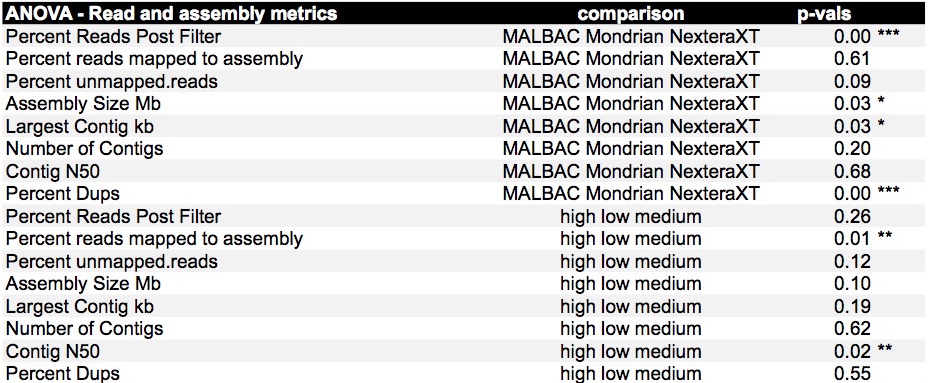


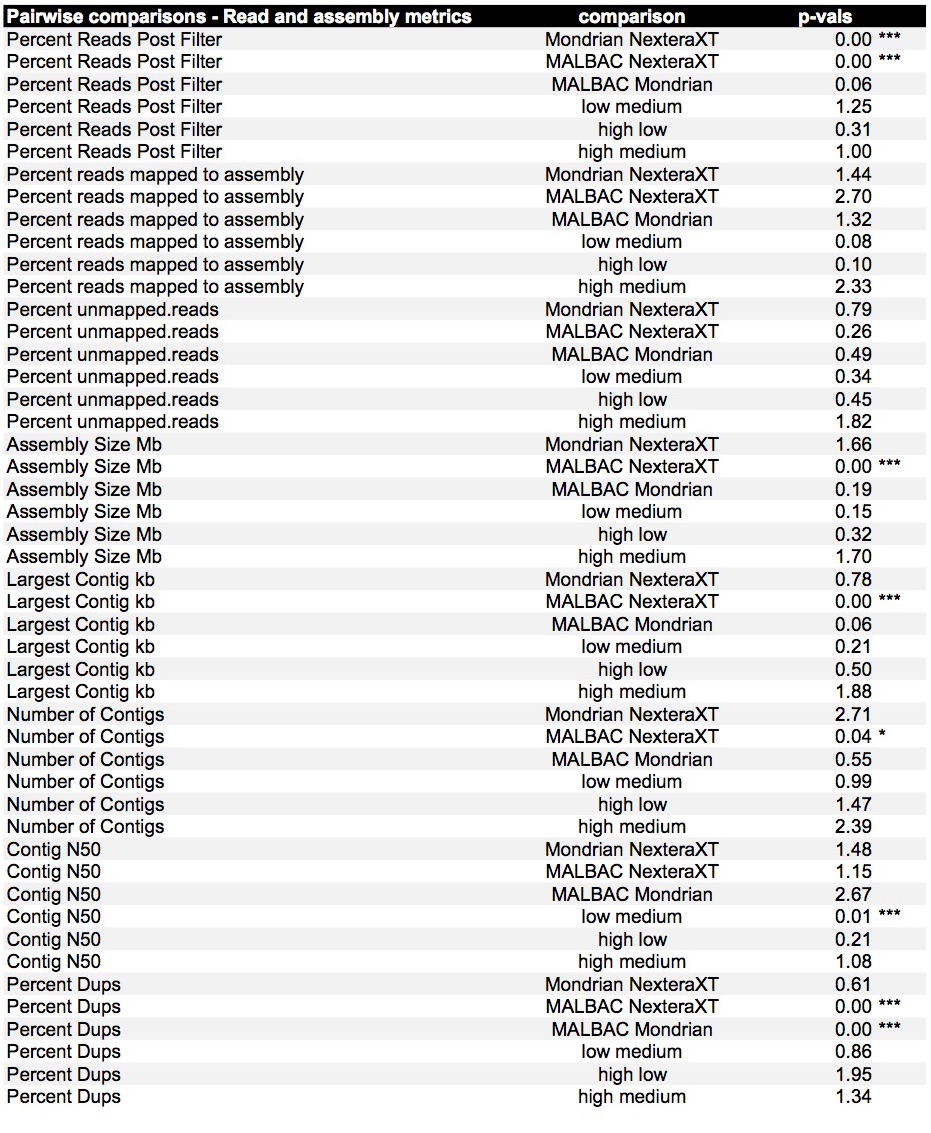

Supplement: Additional file 1: — Supplementary Info. (DOCX 3.22 MB) [file 12864_2015_2063_MOESM1_ESM.docx]
